# Region-specific Risk Factors for Pelvic Lymph Node Metastasis in Patients with Stage IB1 Cervical Cancer

**DOI:** 10.7150/jca.53215

**Published:** 2021-03-05

**Authors:** Jing Zhao, Jing Cai, Hongbo Wang, Weihong Dong, Yuan Zhang, Shaohai Wang, Xiaoqi He, Si Sun, Yuhui Huang, Bangxing Huang, Kay C. Willborn, Ping Jiang, Zehua Wang

**Affiliations:** 1 Department of Obstetrics and Gynecology, Union Hospital, Tongji Medical College, Huazhong University of Science and Technology, Wuhan 430022, China.; 2 Department of Pathology, Union Hospital, Tongji Medical College, Huazhong University of Science and Technology, Wuhan 430022, China.; 3 University Clinic for Medical Radiation Physics, Medical Campus Pius-Hospital, Carl von Ossietzky University Oldenburg, Germany.

**Keywords:** cervical cancer, lymph node metastasis, lymphadenectomy, early stage.

## Abstract

**Objectives:** We aimed to identify the risk factors associated with pelvic lymph node metastasis (LNM) at each anatomic location in patients with stage IB1 cervical cancer.

**Methods:** A primary cohort of 728 patients with stage IB1 cervical cancer who underwent radical hysterectomy and systematic pelvic lymphadenectomy were retrospectively studied. All removed pelvic nodes (*N*=20,134) were pathologically examined. The risk factors for LNM in different anatomic regions (obturator, internal iliac, external iliac, and common iliac) were evaluated by multivariate logistic regression analyses. Nomograms were generated from the primary cohort and validated in another external cohort *(N*=242). The performance of the nomogram was assessed by its calibration and discrimination. Overall survival and progression-free survival in patients with different LNM patterns were compared.

**Results**: LNM was found in 266 (1.3%) removed nodes and 106 (14.6%) patients. The incidences of LNM at the obturator, internal iliac, external iliac, common iliac, and parametrial regions were 8.5%, 5.4%, 4.7%, 1.9% and 1.8%, respectively. Among others, tumour size and lymph-vascular space invasion (LVSI), which are preoperatively assessable, were identified as independent risk factors of LNM in the common iliac region and the lower pelvis, respectively, and age was an additional independent risk factor of obturator LNM. The negative predictive values of tumour size <2 cm for common iliac LNM and negative LVSI combined with older age (> 50 years) for obturator LNM were 100% and 98.7%, respectively. A nomogram of these two factors showed good calibration and discrimination (concordance index, 0.761 in the primary cohort and 0.830 in validation cohort). The patients with common iliac LNM had poorer survival than those with LNM confined to the lower pelvis, while the differences in survival between patients with LNM confined to one node, one region or single side and those with more widely spreading LNM were not statistically significant.

**Conclusions:** Tumour size, LVSI and age are region-specific risk factors for pelvic LNM in IB1 cervical cancer, which could be used to allocate the appropriate extent of pelvic lymphadenectomy.

## Introduction

Cervical cancer is one of the most frequent malignancies in women. It was estimated that 569,847 new cases of cervical cancer were diagnosed and that 311,365 women died of this disease globally in 2018 1. With the rapid development of diagnostic techniques and the widespread use of screening tests, an increasing number of patients with cervical cancer are diagnosed at an early stage. Radical hysterectomy combined with pelvic lymphadenectomy is the first choice of treatment for early stage cervical cancer. A relatively good oncological outcome can be expected in these patients, with a 5-year progression-free survival (PFS) of 88% - 96% 2, 3. However, some cured patients suffer from lifelong treatment complications, which may include severe ureteral and bladder lesions, sexual dysfunction, nerves and blood vessels injuries, and anorectal mobility disorders 4-6. Reducing the treatment-associated adverse effects is a current challenge.

Lymph node metastasis (LNM) is one of the most critical prognostic factors for survival in patients with early-stage cervical cancer, which determines the necessity of adjuvant radiotherapy. To assess the LNM status accurately, a systematic pelvic lymphadenectomy involving nodes in obturator, internal iliac, external iliac, and common iliac regions is routinely performed 7. However, this surgical procedure could lead to complications such as injuries of vessels, nerves and ureters, lower extremity lymph oedema and pelvic lymphocele, impacting the long-term quality of life of patients 8-11. The extent of lymphadenectomy and the number of removed nodes were significant risk factors for these complications 12. The urgent need for a less radical procedure for lymph node staging is the major reason for the surge in the use of sentinel lymph node (SLN) mapping and biopsy worldwide 13. This technique benefits patients by abandoning pelvic lymphadenectomy and decreasing surgical morbidities. The bilateral SLN detection rate and the sensitivity of SLN mapping for early stage cervical cancer were approximately 80% and 90%, respectively 14. That means that about 20% patients need a bilateral or unilateral pelvic lymphadenectomy after SLN and 10% patients are at risk for missed diagnosis of LNM. On the other hand, over 80% of cervical cancers occur in low-resource countries, where the SLN technique is unavailable or not widely used 15. Thus, even in the SLN era, a preoperative evaluation of the risk for pelvic LNM at different anatomic regions to determine the appropriate extent of lymphadenectomy remains meaningful.

Although the risk factors for pelvic LNM have been widely investigated 16-18, we know little about whether their predictive values for LNM are similar or different across all the pelvic lymph node groups. In this study, we retrospectively analyzed the pelvic LNM in 728 patients with stage IB1 (FIGO 2009) cervical cancer, attempting to identify the independent risk factors for metastasis to lymph nodes at different anatomic regions, which might help us optimize the extent of pelvic lymphadenectomy with or without SLN mapping.

## Materials and methods

The study protocol was approved by the Ethics Committee of Tongji Medical College, Huazhong University of Science and Technology (No:2019-S1238). Inclusion criteria: (1) patients who were pathologically confirmed as primary cervical cancer; (2) staged as IB1 based on physical examination combined with imaging according to FIGO 2009; and (3) underwent laparoscopic or open radical hysterectomy and systematic pelvic lymphadenectomy from January 2008 to December 2019 at Union Hospital, Wuhan, China. The exclusion criteria were: (1) the patients had other malignant tumours; (2) preoperative radiotherapy or chemotherapy; and (3) no detailed records of regional lymph node resection and metastasis. The patients underwent surgery from January 2008 to December 2017 were studied as the primary cohort (N=728) to delineate the pelvic LNM pattern and as the model development cohort to identify the risk factors for LNM, and those from January 2018 to December 2019 were allocated to validation cohort to validate the model performance (*N*=242).

The surgical procedure was performed according to the C type of the Querleu-Morrow classification 19, as described in our previous article 20. Systematic bilateral pelvic lymphadenectomy included the dissection of the nodes in the common iliac, external iliac, internal iliac, and obturator regions, and parametric lymph nodes were removed through radical hysterectomy. The cranial boarder of the lymphadenectomy was the level of the inferior mesenteric artery, the lateral border was the genitofemoral nerve, and the caudal border was the pelvic floor. Anatomic landmarks of the specified regions are shown in Table 1.

For pathological examinations, the nodes in fat tissue were detected by palpation, which allowed nodes larger than 3 mm in diameter to isolate. When the number of the isolated nodes detected by palpation were less than 25, the rest of the fat tissue would be also paraffin-embedded for evaluation of smaller lymph nodes. Generally, three sections were examined for each node. Parametrial nodes were identified in the giant sections of radical hysterectomy specimens.

All statistical analyses were performed using the IBM SPSS Statistics 22.0 software package (SPSS Inc., Chicago, Illinois) and R software (version 4.0.3). The risk factors for pelvic LNM were evaluated by univariate and multivariate logistic regression analyses with odds ratios and 95% confidence intervals (CI). The significant factors revealed by the univariate analyses were included in the multivariate regression analyses. And then nomograms based on the multivariate analysis was constructed and validated in the validation cohort. To assess the discrimination and the calibration performance of the nomogram model, the concordance index was measured by calculating the area under the receiver operating characteristics curve (ROC) and the calibration curves were plotted. In addition, the Hosmer-Lemeshow test was used to judge the fit performance of the model. Survival curves for overall survival (OS) and PFS were plotted by the Kaplan-Meier method and compared by the log-rank test. A significance level of 0.05 was used in all tests.

## Results

### Overall pelvic LNM

There were 728 women with stage IB1 cervical cancer included in the present study. With 27.80 ± 9.43 lymph nodes removed per patient, a total of 20,134 lymph nodes were obtained from the obturator, internal iliac, external iliac, common iliac, and parametrial regions of these patients and pathologically examined. LNM was observed in 106 (14.6%) women. Positive nodes were found in 6.7% (17/254) of patients with tumours smaller than 2 cm in diameter, while the incidence was 19.5% (82/421) in those with larger tumours. The characteristics of the patients and their tumours are summarized in Table 2. The potential clinicopathological risk factors associated with LNM were evaluated by univariate and multivariate regression analyses. As shown in Table 3, a significantly increased risk of LNM was present in patients with LVSI (OR, 5.14; *P* < 0.001), parametrial invasion (OR, 2.98; *P* = 0.011), and tumour size ≥ 2 cm (OR, 2.08; *P* = 0.017), while age, pathological type, histological grading and number of removed nodes were not independent risk factors for LNM.

### Distribution of positive pelvic lymph nodes

**Figure 1:**
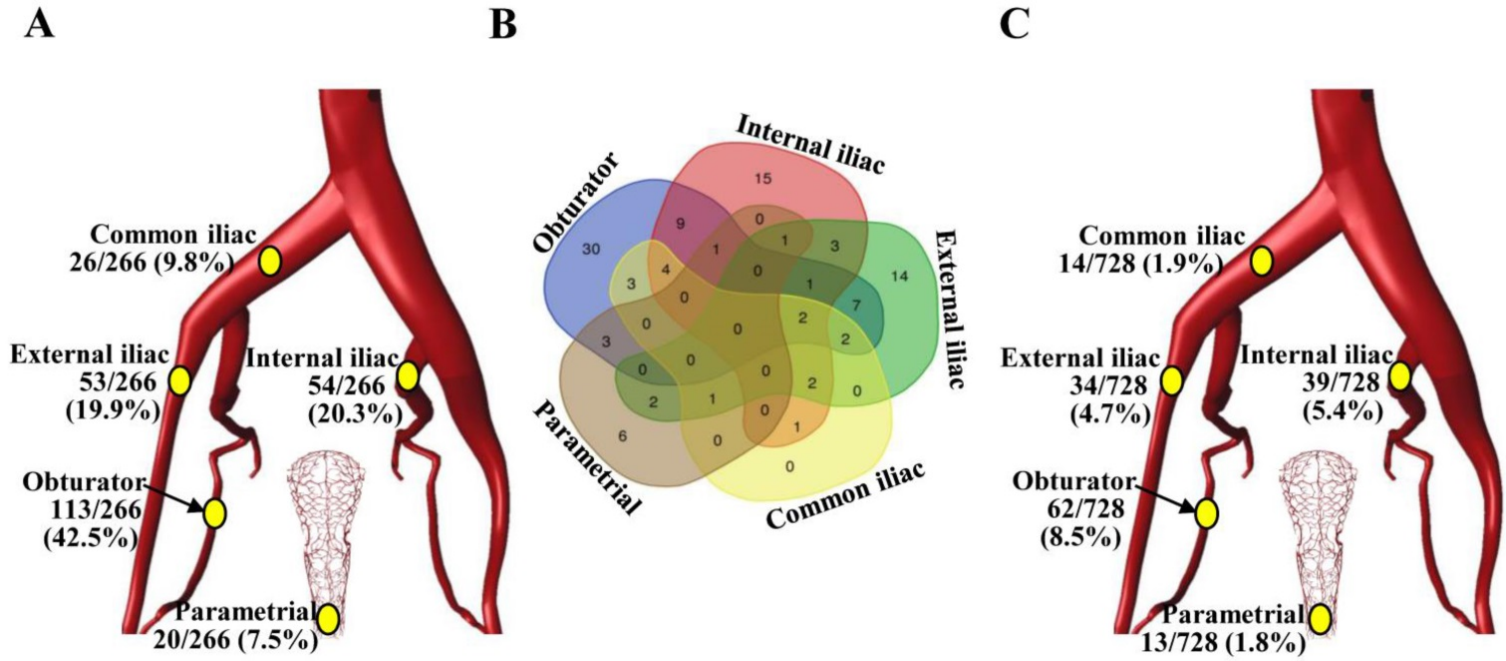
The incidence and distribution of pelvic lymph node metastasis in patients with stage IB1 cervical cancer. **(A)** Distribution of the 266 positive lymph nodes. **(B)** Venn diagram showing the number of patients with lymph node metastasis in different regions. **(C)** The incidence of pelvic lymph node metastasis at individual anatomic regions in 728 patients.

Among the 20,134 lymph nodes removed, 266 (1.3%) showed metastatic cancer deposits. The obturator was the most common site for nodal metastasis (113/266, 42.5%), followed by the internal iliac nodes (54/266, 20.3%) and the external iliac nodes (53/266, 19.9%), while the common iliac (26/266, 9.8%) and parametrial (20/266, 7.5%) nodes were the least likely to be involved (Figure 1A). Among the 106 patients with LNM, 50 (47.2%) women had a single positive node, 25 (23.6%) had two positive nodes, 13 (12.3%) had three positive nodes, and 18 (17.0%) had four or more positive nodes. Unilateral LNM and bilateral LNM were found in 64.2% (68/106) and 35.8% (38/106) of patients, respectively. In 61.3% (65/106) of patients, lymph node metastases were confined to a single region, particularly the obturator region (*N*=30), while isolated common iliac LNM was not observed (Figure 1B). The incidences of LNM at the obturator, internal iliac, external iliac, common iliac, and parametrial regions in the entire cohort (*N*=728) were 8.5%, 5.4%, 4.7%, 1.9% and 1.8%, respectively (Figure 1C).

### Risk factors associated with LNM at different anatomic regions

Next, the association of risk factors for lymphatic metastasis in specific anatomic locations was further investigated. For LNM at the common iliac nodes, tumour size and parametrial invasion were independent risk factors. All the positive common iliac nodes were found in patients with tumours greater than 2 cm, and the multivariate analysis showed that tumour size of greater than 3 cm was associated with a 16.6-fold increase in the risk for common iliac LNM (Table 4). Interestingly, tumour size was not an independent risk factor for pelvic LNM in the lower regions, i.e., the obturator, internal iliac and external iliac areas, where LVSI was the most significant predictor for LNM. In addition, parametrial invasion was related to external and internal iliac LNM; deep stromal invasion and age of less than 50 years were associated with obturator LNM (Table 5 and Table S1). Among the risk factors of obturator LNM, age and LVSI are available preoperatively. In patients younger than 50 years with LVSI, the incidence of obturator LNM was 25.4% (33/130), with a 6.68-fold increased risk (95% CI, 3.88-11.49) compared to other patients (*P*<0.001). The combination of age ≥ 50 years and negative LVSI had a negative predictive value of 98.7% (155/157) for obturator LNM.

### Survival outcomes associated with nodal metastasis patterns

**Figure 2:**
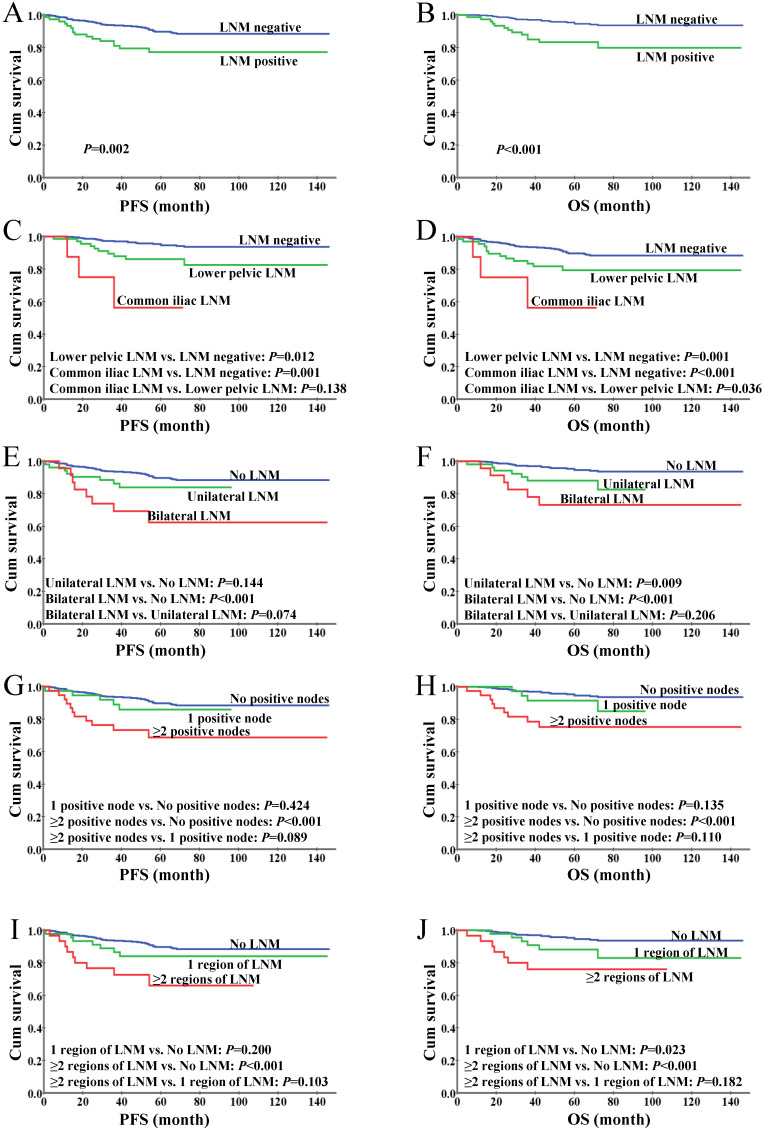
Kaplan-Meier analysis of survival for IB1 cervical cancer patients. **(A)** Progression-free survival based on the status of lymph nodes. **(B)** Overall survival based on the status of lymph nodes. **(C)** Progression-free survival stratified by LNM anatomic regions. **(D)** Overall survival stratified by LNM anatomic regions. **(E)** Progression-free survival stratified by unilateral and bilateral LNM. **(F)** Overall survival stratified by unilateral and bilateral LNM. **(G)** Progression-free survival based on the number of positive lymph nodes. **(H)** Overall survival based on the number of positive lymph nodes. **(I)** Progression-free survival based on the number of regions of LNM. **(J)** Overall survival based on the number of regions of LNM.

With a median follow-up time of 67 months (range, 5-146), we found a 5-year OS of 92.9% and a 5-year PFS of 87.9% in the entire cohort. The 5-year OS and PFS rates were significantly decreased in the patients with LNM when compared to those with negative nodes (84.7% vs. 94.2%, *P*=0.01; 74.7% vs. 89.6%, *P*<0.01; Figure 2A-B). The patients with LNM involving the common iliac region had a significantly worse OS than those with LNM confined to lower pelvic nodes (*P*=0.036); a similar trend was observed in PFS but with no statistical significance (*P*=0.138, Figure 2C-D), which might due to the limited sample size of the common iliac LNM group (*N*=8) and the short follow-up period (up to 57 months). The differences in survival between patients stratified by unilateral versus bilateral LNM, single one versus more than one positive node and single region versus more than one region of LNM were not statistically significant (Figure 2E-J), while the patients with even only one positive node showed significantly inferior OS and PFS versus those with no LNM, suggesting the presence of LNM rather than its extent is a determinant of the oncological outcomes in patients with early-stage cervical cancer.

### Nomograms construction and validation

**Figure 3:**
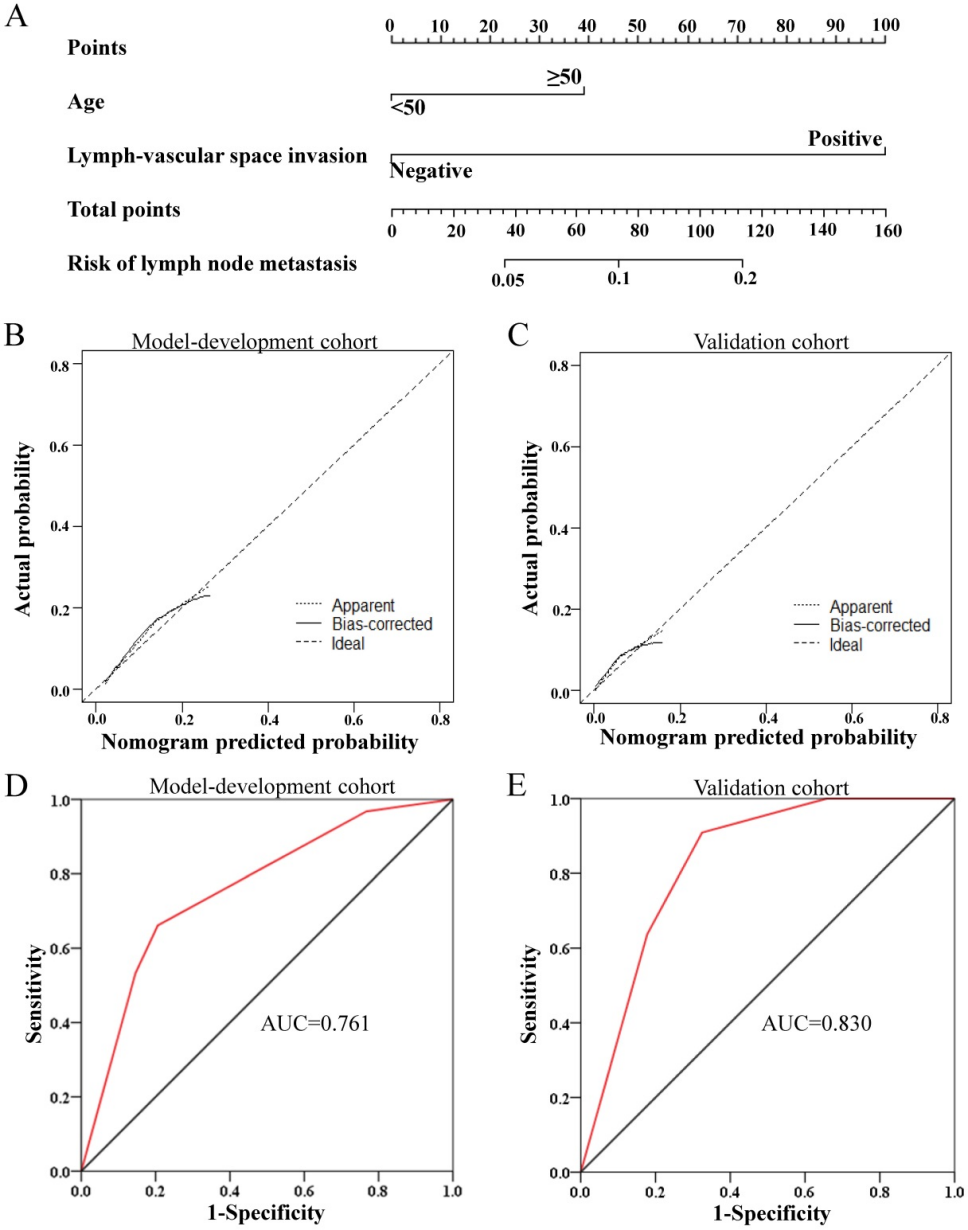
Development and performance of the nomogram C for obturator lymph node metastasis. **(A)** The nomogram C was developed in the model development cohort, with lymph-vascular space invasion and age. **(B, C)** Calibration curves of the nomogram C in the model development cohort (B) and validation cohorts (C). **(D, E)** ROC plots of the nomogram in the model development cohort (D, AUC=0.761, 95% CI=0.699-0.823) and validation cohorts (E, AUC=0.830, 95% CI=0.743-0.918)

Nomograms were constructed based on the multivariate logistic regression analysis in primary cohort (*N*=728) and validated in the external validation cohort (*N*=242). The clinicopathological characteristics of the patients in validation cohort were shown in Table S2. For overall pelvic LNM, a nomogram A was generated based on the multivariate logistic regression model composed of PI, LVSI and tumour size, as shown in Figure S1. This nomogram model yielded a concordance index of 0.774 (95% CI, 0.723 - 0.825) in the model development cohort and 0.854 (95% CI, 0.794 - 0.915) in the validation cohort, demonstrating good agreement between prediction and observation in the two cohorts. In addition, the Hosmer-Lemeshow test proved the nomogram A was well-fitted with a *P* value of 0.785. Considering that PI could not be evaluated accurately before surgery, a nomogram B based only on the other two independent risk factors known before surgery was built and validated additionally, also showing good discrimination and calibration (Figure S2). Moreover, for obturator LNM, the most common LNM, a nomogram C was constructed based on LVSI and age, which could be known before surgery, and validated in the validation cohort, with a concordance index of 0.761 (95% CI, 0.699-0.823) and 0.830 (95% CI, 0.743-0.918) in the primary cohort and external validation cohort, respectively (Figure 3). In addition, Hosmer-Lemeshow test proved the nomogram was well-fitted with a *P* value of 0.616.

## Discussion

Although the risk factors of LNM in cervical cancer have been well studied, there are limited evidence on the specific risk factors of LNM at different autonomic regions in pelvis. In the present study, we delineated the pelvic LNM pattern in 728 patients with stage IB1 cervical cancer and found that, among others, larger tumour size and LVSI are specifically associated with LNM in the common iliac area and the low true pelvis, respectively, in patients with stage IB1 cervical cancer. These findings provide a fundamental basis for optimizing the treatment regarding lymph node dissection to reduce complications without compromising oncological outcomes.

We found an LNM incidence of 14.6% in patients with IB1 cervical cancer, which is identical with the data previously reported 21, 22. As neither SLN mapping nor ultrastaging had been performed in these patients, micrometastases are not to be excluded in those N0 cases, which is considered a negative factor for survival and an indication for adjuvant chemotherapy after surgery 23. Fortunately, the 5-year PFS rate in this subgroup reached 94.2%, which might due to the low incidence of micrometastasis in cases without macrometastasis 24. As to the topographic distribution of positive pelvic nodes, the obturator, internal iliac and external iliac were the most prevalent sites with a metastasis rate of 8.5%, 5.4%, and 4.7%, respectively. A previous study including 189 patients with IB1 cervical cancer revealed similar LNM rates in the obturator (9.5%) and internal iliac (4.9%) but a lower rate in the external iliac (1.7%) 25. This inconsistence could be explained by the disparity in the nomenclature of lymph node groups; the lymph nodes caudal to the deep circumflex iliac vessels were included in the external iliac in our study.

Among the known risk factors of LNM in patients with early stage cervical cancer, tumour size and LVSI are assessable before surgery through imaging and histological evaluation of specimens of conisation, loop electrical excision procedure, and cervical biopsy, respectively 26, 27. Interestingly, in the present study, LVSI was identified as an independently predictive for metastasis in all three lower groups of pelvic lymph nodes but not in common iliac LNM; on the contrary, tumour size was a robust predictor for common iliac LNM but not significantly associated with lower pelvic node metastasis. Given the extremely high negative prediction values (100% and 98.7%) of smaller tumour size (<2 cm) for common iliac LNM and the combination of negative LVSI and older age (> 50 years) for obturator LNM, these regions could be omitted in pelvic lymphadenectomy in patients with relevant low-risk features. Nevertheless, the oncological safety of these adjustments in lymphadenectomy needs to be proven in randomized trials.

Over the past two decades, emerging findings suggest that SLN mapping and biopsy could be an alternative to systematic pelvic lymphadenectomy used to stage LNM with acceptable accuracy through a minimized removal of lymph nodes 28, 29. The most common localisations of SLNs are the obturator, internal iliac and external iliac areas 30, being consistent with the frequent metastatic sites shown in the present study. However, there are concerns about false negative results revealed by SLN mapping, especially in tumours larger than 2 cm 31. Thus, the SLN technique is mainly recommended for patients with smaller tumour, and ultrastaging based on immunohistochemistry is used to improve the detection of micrometastasis in SLNs 32. Despite the advances in SLN biopsy, false negative rates of intraoperative SLN evaluation ranging from approximately 30% to 80% have been reported 31, 33, 34. Our findings support that, for tumours greater than 2 cm, systematic lymphadenectomy would be safer in terms of lymph node staging than SLN mapping considering the increased risk of metastasis in the common iliac nodes. Moreover, for patients with positive LVSI but negative SLN biopsy, especially when they are younger than 50 years old, additional removal of the lower pelvic nodes would minimize the risk of missed diagnosis of LNM.

In the present study, we identified and validated the region-specific risk factors of pelvic LNM in a large-scale cohort of patients with IB1 cervical cancer undergoing systematic pelvic lymphadenectomy. The mean number of removed lymph nodes of 27.80 indicates the radicalness of the surgical procedure 35, providing reliable data regarding topographic distribution of LNM for further analysis of site-specific risk factors. However, the single-centric, retrospective study design might cause bias in the results. Moreover, only IB1 tumours are included and the results might be difficult to extrapolate to tumours in other early stages because of varied pathological features and LNM incidence across different stages. Randomized studies are needed to investigate the potential benefits and risks of incorporating our results in less radical lymphadenectomy strategy or in SLN mapping algorithm.

In conclusion, tumour size and LVSI, as independent risk factors for common iliac and lower pelvic LNM, respectively, could be used to allocate the appropriate extent of pelvic lymphadenectomy (with or without SLN mapping) for patients with IB1 cervical cancer.

## Supplementary Material

Supplementary figures and tables.

## Figures and Tables

**Table 1:** Anatomic Landmarks of the specified area.

Lymph nodes regions	Anatomic landmarks
Common iliac	The lymph nodes between the bifurcation level of the aorta and the bifurcation of the iliac vessels.
External iliac	The lymph nodes along the external iliac vessels, including the lymph nodes caudal to the deep circumflex iliac vessels.
Internal iliac	The lymph nodes medial to the internal iliac vessel down to the level of the bifurcation of the uterine vessels.
Obturator	The lymph nodes in the obturator fossa (between the external and internal iliac).
Parametrial	The lymph nodes in parametrium, which are removed as a part of radical hysterectomy.

**Table 2:** Clinicopathological characteristics of patients (*N*=728).

Variables	Number of patients (%)	Patient with LNM (%)
Age (years)		
Median (range)	45 (25-76)	
<50	523 (71.8)	83 (15.9)
≥50	205 (28.2)	23 (11.2)
Tumor size		
<2 cm	254 (34.9)	17 (6.7)
≥2 cm	421 (57.8)	82 (19.5)
Unknown	53 (7.3)	7 (13.2)
Stromal invasion		
Inner 1/3	189 (26.0)	14 (7.4)
Middle 1/3	104 (14.3)	19 (18.3)
Outer 1/3	241 (33.1)	59 (24.5)
Unknown	194 (26.6)	14 (7.2)
LVSI		
Absent	550 (75.5)	43 (7.8)
Present	178 (24.5)	63 (35.4)
Parametrial invasion		
Absent	698 (95.9)	90 (12.9)
Present	30 (4.1)	16 (53.3)
Pathologic type		
Squamous cell cancer	566 (77.7)	90 (15.9)
Adenocarcinoma	146 (20.1)	14 (9.6)
Others	16 (2.2)	2 (12.5)
Histologic grading		
Well differentiated, G1	108 (14.8)	6 (5.6)
Moderately differentiated, G2	371 (51.0)	58 (15.6)
Poorly differentiated, G3	232 (31.9)	41 (17.7)
Unknown	17 (2.3)	1 (5.9)
Removed LN		
Mean± SD	27.80±9.43	
<30	462 (63.5)	63 (13.6)
≥30	266 (36.5)	43 (16.2)

LNM, Lymph node metastasis; LVSI, lymphovascular space involvement; LN, lymph nodes; SD, Standard deviation.

**Table 3:** Analysis of the risk factors for pelvic lymph node metastasis in patients with IB1 cervical cancer (*N*=728).

Variables	Univariate regression analyses			Multivariate regression analyses		
	OR	95% CI	*P*	OR	95% CI	*P*
PI, positive vs. negative	7.72	3.65-16.36	<0.001	2.98	1.29-6.92	0.011
LVSI, positive vs. negative	6.46	4.17-10.00	<0.001	5.14	3.14-8.42	<0.001
Tumor size, ≥2 cm vs. <2 cm	3.37	1.95-5.83	<0.001	2.08	1.14-3.79	0.017
DSI, positive vs. negative	2.99	1.95-4.57	<0.001	1.44	0.87-2.38	0.158
Grading, G2-3 vs. G1	3.34	1.43-7.82	0.005	1.71	0.69-4.26	0.249
Age, ≥50 y vs. <50 y	0.67	0.41-1.10	0.111	-	-	-
Removed LN, ≥30 vs. <30	1.22	0.80-1.86	0.352	-	-	-
Pathologic type, AC vs. SC	0.56	0.31-1.02	0.057	-	-	-

PI, parametrial invasion; LVSI, lymphovascular space involvement; DSI, deep stromal invasion; LN, lymph nodes; AC, Adenocarcinoma; SC, Squamous cell cancer; OR, odds ratio; CI, confidence interval.

**Table 4:** Analyses of the risk factors for lymphatic metastasis in common iliac lymph nodes.

Variables	Univariate regression analyses			Multivariate regression analyses		
	OR	95% CI	*P*	OR	95% CI	*P*
PI, Positive vs. Negative	15.31	4.78-49.03	<0.001	4.84	1.37-17.15	0.015
LVSI, Positive vs. Negative	4.27	1.46-12.47	0.008	2.13	0.66-6.87	0.206
Tumor size, ≥3 cm vs. <3 cm	24.20	3.15-186.15	0.002	16.60	2.10-131.41	0.008
DSI, Positive vs. Negative	2.60	0.89-7.58	0.080	-	-	-
Age, ≥50 y vs. <50 y	0.19	0.03-1.48	0.113	-	-	-
Removed LN, ≥30 vs. <30	0.96	0.32-2.91	0.948	-	-	-
Pathologic type, AC vs. SC	0.64	0.14-2.90	0.564	-	-	-
Grading, G2-3 vs. G1	2.36	0.31-18.21	0.411	-	-	-

PI, parametrial invasion; LVSI, lymphovascular space involvement; DSI, deep stromal invasion; LN, lymph nodes; AC, Adenocarcinoma; SC, Squamous cell cancer; OR, odds ratio; CI, confidence interval.

**Table 5:** Multivariate regression analyses of the risk factors for lymphatic metastasis in obturator, internal iliac, and external iliac lymph nodes.

Variables	External iliac nodes			Obturator nodes			Internal iliac nodes		
	OR	95% CI	*P*	OR	95% CI	*P*	OR	95% CI	*P*
PI, positive vs. negative	3.83	1.45-10.16	0.007	2.29	0.92-5.73	0.076	2.79	1.02-7.62	0.045
LVSI, positive vs. negative	4.84	2.20-10.66	<0.001	6.39	3.43-11.90	<0.001	3.68	1.75-7.73	0.001
Tumor size, ≥2 cm vs. <2cm	1.80	0.83-3.89	0.135	1.34	0.72-2.5q	0.359	1.28	0.60-2.72	0.529
DSI, positive vs. negative	-	-	-	2.14	1.00-4.55	0.049	2.10	0.82-5.39	0.123
Age, ≥50 y vs. <50 y	-	-	-	0.37	0.17-0.80	0.012	-	-	-

PI, parametrial invasion; LVSI, lymphovascular space involvement; DSI, deep stromal invasion; OR, odds ratios; CI, confidence interval.

## Abbreviations

LNM: lymph node metastasis
PI: parametrial invasion
LVSI: lymphovascular space involvement
DSI: deep stromal invasion
SLN: sentinel lymph node
PFS: progression-free survival
OS: overall survival
