# Supplementary material for: Region-specific Risk Factors for Pelvic Lymph Node Metastasis in Patients with Stage IB1 Cervical Cancer
Source: J Cancer. 2021 Mar 5;12(9):2624–32. doi: 10.7150/jca.53215 (PMC8040729; doi:10.7150/jca.53215)
Supplement: Supplementary file 1 — Supplementary figures and tables. [file jcav12p2624s1.pdf]

Table S1. Univariate regression analyses of the risk factors for lymphatic metastasis in obturator, internal iliac, and external iliac lymph nodes.

Variables	External iliac nodes			Obturator nodes			Internal iliac nodes		
	**OR**	**95% CI**	***P***	**OR**	**95% CI**	**P**	**OR**	**95% CI**	***P***
PI, positive vs. negative	9.40	3.83-23.09	<0.001	6.21	2.76-13.96	<0.001	6.33	2.53-15.85	<0.001
LVSI, positive vs. negative	7.27	3.47-15.25	<0.001	7.54	4.31-13.18	<0.001	4.42	2.29-8.53	<0.001
Tumor size, ≥2 cm vs. <2 cm	3.28	1.61-6.68	0.001	2.28	1.34-3.86	0.002	2.79	1.44-5.42	0.002
DSI, positive vs. negative	1.94	0.86-4.37	0.109	3.29	1.64-6.61	0.001	3.17	1.30-7.73	0.011
Age, ≥50 y vs. <50 y	0.65	0.28-1.52	0.318	0.46	0.23-0.93	0.031	0.87	0.42-1.83	0.719
Removed LN, ≥30 vs. <30	0.47	0.18-1.23	0.124	0.88	0.51-1.52	0.649	0.97	0.50-1.90	0.932
Pathologic type, AC vs. SC	0.82	0.33-2.03	0.673	0.65	0.31-1.35	0.248	0.32	0.10-1.05	0.060
Grading, G2-3 vs. G1	-	-	0.996	1.74	0.73-4.15	0.211	3.37	0.80-14.19	0.098

PI, parametrial invasion; LVSI, lymphovascular space involvement; DSI, deep stromal invasion; HG, Histologic grading ; LN, lymph nodes; AC, Adenocarcinoma; SC, Squamous cell cancer; OR, odds ratios; CI, confidence interval.

Table S2. Clinicopathological characteristics of the patients in validation cohort (*N*=242).

**Variables**	**Number of patients (%)**	**Patient with LNM (%)**
Age (years)		
Median (range)	49 (26-68)	
<50	126 (52.1)	16 (12.7)
≥50	116 (47.9)	6 (5.2)
Tumor size		
<2 cm	126 (52.1)	5 (4.0)
≥2 cm	116 (47.9)	17 (14.7)
Stromal invasion		
Inner 1/3	120 (49.6)	3 (2.5)
Middle 1/3	70 (28.9)	7 (10.0)
Outer 1/3	52 (21.5)	12 (23.1)
LVSI		
Absent	157 (64.9)	2 (1.3)
Present	85 (35.1)	20 (23.5)
Parametrial invasion		
Absent	238 (98.3)	19 (8.0)
Present	4 (1.7)	3 (75.0)
Pathologic type		
Squamous cell cancer	176 (72.7)	21 (11.9)
Adenocarcinoma	60 (24.8)	1 (1.7)
Others	6 (2.5)	0 (0)
Histologic grading		
Well differentiated, G1	13 (5.4)	0 (0)
Moderately differentiated, G2	139 (57.4)	17 (12.2)
Poorly differentiated, G3	62 (25.6)	5 (8.1)
Unknown	28 (11.6)	22 (10.3)
Removed LN		
Mean ± SD	36.80 ± 10.63	
<30	63 (26.0)	7 (11.1)
≥30	179 (74.0)	15 (8.4)

LNM, Lymph node metastasis; LVSI, lymphovascular space involvement; LN, lymph nodes; SD, Standard deviation.

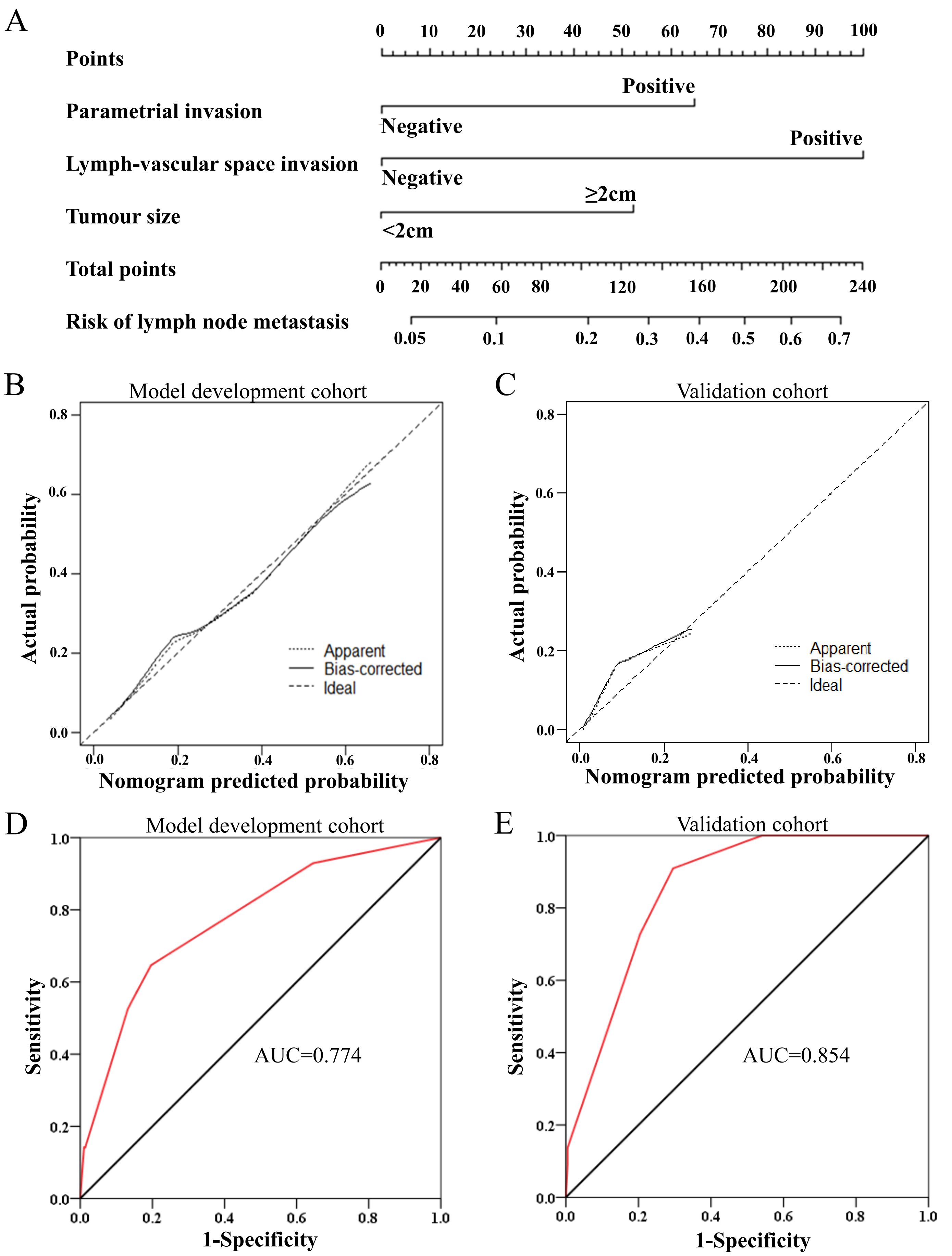

Figure S1. Development and performance of nomogram A. (A) The nomogram A was developed in the model development cohort, with parametrial invasion, lymph-vascular space invasion and tumour size. (B, C) Calibration curves of the nomogram in the model development cohort (B) and validation cohorts (C). (D, E) ROC plots of the nomogram A in the model development cohort (D, AUC=0.774, 95% CI=0.723-0.825) and validation cohorts (E, AUC=0.854, 95% CI=0.794-0.915)

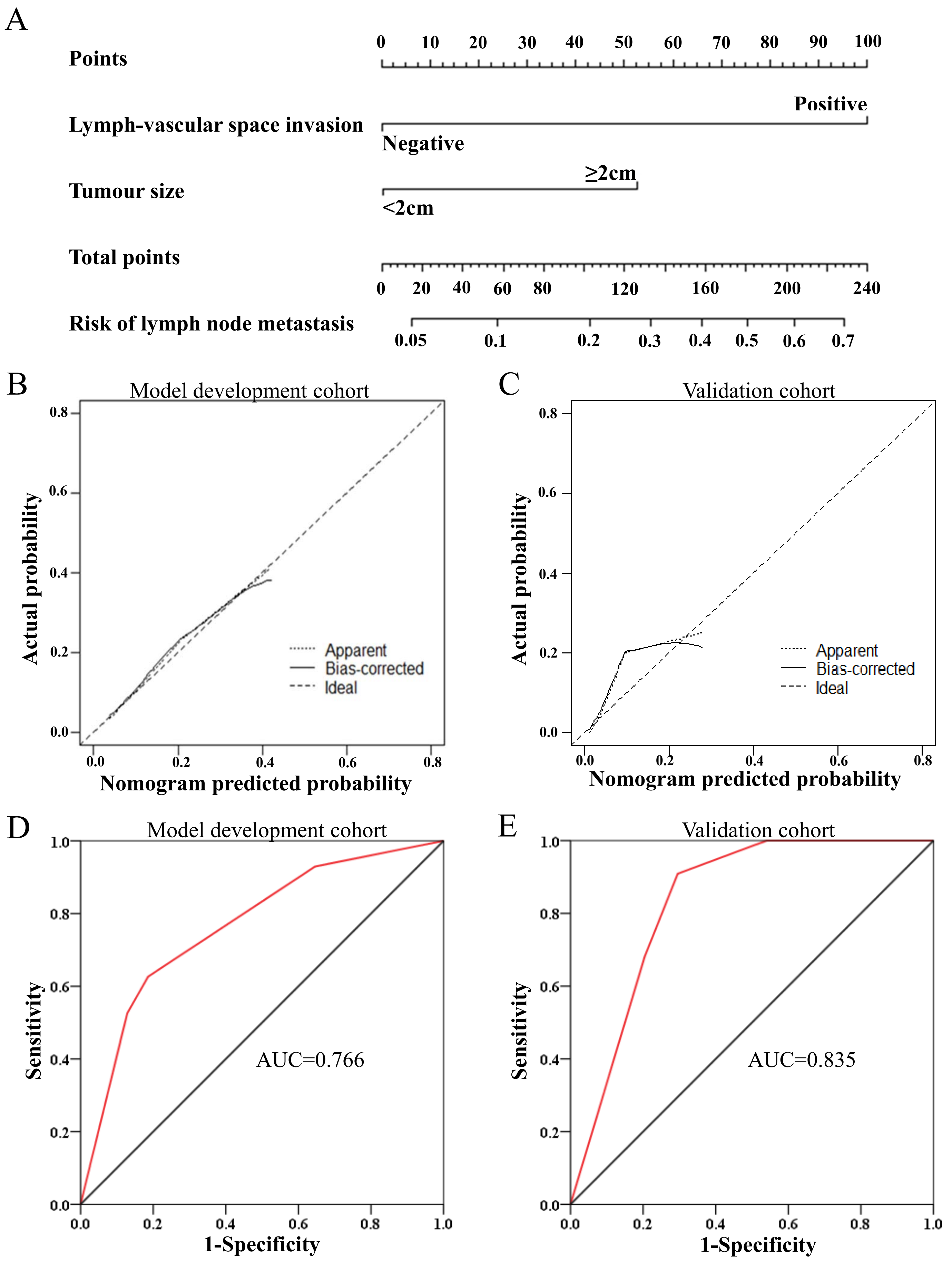

Figure S2. Development and performance of nomogram B. (A) The nomogram B was developed in the model development cohort, with lymph-vascular space invasion and tumour size. (B, C) Calibration curves of the nomogram in the model development cohort (B) and validation cohorts (C). (D, E) ROC plots of the nomogram in the model development cohort (D, AUC=0.766, 95% CI=0.715-0.817) and validation cohorts (E, AUC=0.835, 95% CI=0.775-0.915)
